# Behavioral Analysis of EEG Signals in Loss-Gain Decision-Making Experiments

**DOI:** 10.1155/2022/3070608

**Published:** 2022-07-15

**Authors:** Jiaquan Shen, Ningzhong Liu, Deguang Li, Binbin Zhang

**Affiliations:** ^1^School of Information Science, Luoyang Normal University, Luoyang 471022, China; ^2^College of Computer Science and Technology, Nanjing University of Aeronautics and Astronautics, Jiangsu Nanjing 211106, China

## Abstract

Extraction and analysis of the EEG (electroencephalograph) information features generated during behavioral decision-making can provide a better understanding of the state of mind. Previous studies have focused more on the brainwave features after behavioral decision-making. In fact, the EEG before decision-making is more worthy of our attention. In this study, we introduce a new index based on the reaction time of subjects before decision-making, called the Prestimulus Time (PT), which have important reference value for the study of cognitive function, neurological diseases, and other fields. In our experiments, we use a wearable EEG feature signal acquisition device and a systematic reward and punishment experiment to obtain the EEG features before and after behavioral decision-making. The experimental results show that the EEG generated after behavioral decision due to loss is more intense than that generated by gain in the medial frontal cortex (MFC). In addition, different characteristics of EEG signals are generated prior to behavioral decisions because people have different expectations of the outcome. It will produce more significant negative-polarity event-related potential (ERP) in the forebrain area when the humans are optimistic about the outcomes.

## 1. Introduction

At present, a series of research results have been obtained by using EEG to study behavioral decision-making [1–3]. There is a more profound recognition about decision-making in neural systems, especially those concerned with rewards and punishments [4, 5]. These studies use the system of rewards and punishment experiments to extract the features in EEG [6–8]. In addition, human behaviors can be influenced more intensively by the change of reward and punishment mechanism, such as conditioned reinforces and probability changes [9–11]. Moreover, people often change their behaviors to avoid monetary losses [12, 13]. In particular, the EEG can be more powerfully influenced by people's expectations, and the different expected values of the outcomes will also have different effects on EEG [14, 15]. In the study of ERP (event-related potential), it was found that the MFN (medial-frontal negativity) is particularly sensitive to the valence of rewards or performances. The EEG in front of the central recording sites reaches maximum negative-polarity ERP between 250 and 300 ms postonset of feedback stimulus [16, 17]. The MFN is more sensitive to negative feedback affected by bad outcomes, such as error responses or pecuniary losses, than to positive feedback [18, 19].

Previous studies on neural activities related to losses and gains are mainly concerned with the outcomes presented. For example, the studies based on scalp recording and neuroimaging have shown that information after the outcomes is presented [20, 21]. The indicators of the study are P300, N400, etc. These ERPs are based on the value of positive or negative and time of the EEG after stimulation. These indicators play an important role in the study of cognitive dysfunction and neurological diseases, which have important reference value. However, most of the previous studies have ignored the reflection of the EEG before the stimulus, especially in the reward and punishment experiments with enhanced reality. In our study, we propose a new indicator named prestimulus time (PT), whose index reflects the activity and response time of the subjects on the EEG before decision-making, which has important reference value for the study of cognitive dysfunction and neurological diseases. Similar to other ERPs, such as P300 and N400, this index can be used as an indicator to reflect cognitive impairment and observe the effects of neuropsychotic treatment. The PT can be used as a supplementary reference for other ERPs. We believe that the PT will be more applied in future researches, especially in the field of cognitive science.

Several findings have shown that in the gain frame, the participants tend to choose more risky options after receiving right anodal/left cathodal transcranial direct current stimulation, whereas in the loss frame, the participants tend to choose more safe options [22, 23]. However, these conclusions are more derived from the psychological or statistical significance, and it has not been well documented in EEG studies. Adaptive decision-making depends on the accurate representations of rewards associated with potential choices, and these representations can be acquired with reinforcement learning (RL) mechanisms, which use the prediction error (the difference between expected and received rewards) as a learning signal to update reward expectations [24, 25], while these experiments have highlighted the role of feedback-related potentials during performance monitoring. In our experiments, we extract and preanalyze the EEG before the decision-making and find that there is a clear distinction with different potential choices in the EEG around 100 ms before the decision-making. The methods and results of our research are more credible for the prediction of behavioral decision-making.

The main purpose of our study is to provide further evidence for the impacts of reward valence, reward magnitudes, and magnitude expectancy upon the EEG in outcome evaluation. Our study not only concerned about the impact of outcomes on the EEG but also paid more attention to the preanalysis before making a decision. Here, we conduct three experiments under different reward magnitudes. In every experiment, we analyze the EEG generated after presenting the outcomes and before making a decision, respectively.

## 2. Materials and Methods

### 2.1. EEG Acquisition and Processing Method

**Figure 1:**
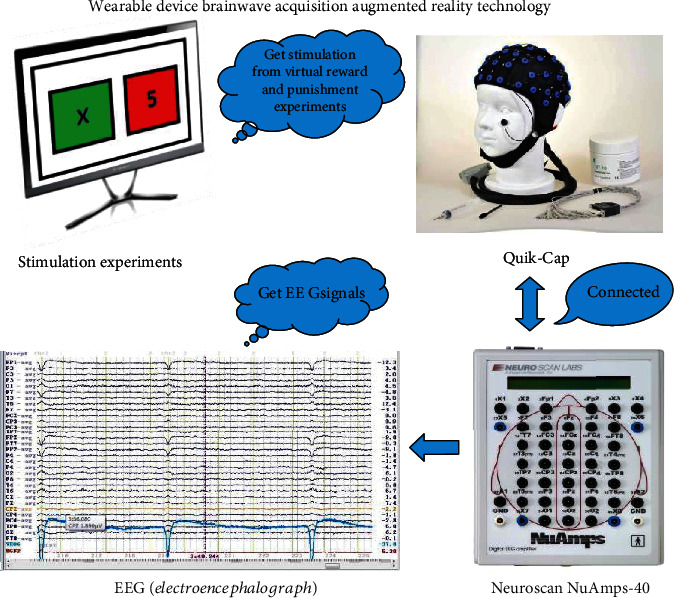
Wearable device EEG acquisition augmented reality technology.

As shown in Figure 1, our experiments adopt wearable device EEG acquisition augmented reality technology. The EEG acquisition devices use NeuroScan40 amplifiers and wearable device electrode caps (Quik-Cap 64) [26–28]. We use Curry-7 software to collect and process EEG signals [29, 30]. The software of Curry-7 can collect EEG signals in higher time domain and frequency domain, which can easily perform denoising, epochs and averaging, principal component analysis, and other operations on collected EEG signals. The stimulation and the choice of the strategies of the subjects are implemented by using psychological experiment software E-prime. The E-prime software can transmit the different decisions selected by the subjects in real time and mark the EEG signals, which will greatly facilitate the differential processing of the EEG signals in the next work.

The process of EEG data is carried out according to the following steps and methods [31, 32]. The data analysis and processing mainly depend on Curry and Matlab.

1. Baseline correction. The baseline correction uses the constant method. This method can perform baseline correction on EEG, which makes the baseline of the waveform coincide with the “*x*-axis” of the label. Before the implementation of this method, the baseline of EEG waveform does not correspond to the label and its amplitude is very large, and the amplitude of many leads is greater than 1000uv. After implementing this method, the baseline of the EEG waveform corresponds to the label and the amplitude is within 100 *μ*v
2. Removal of the effect of ocular electricity on EEG. First, we label the eye electricity. The vertical amplitude is greater than 100 *μ*v, which is caused by blinking; and these waveforms are labeled as ocular electricity. We use the correlation coefficient to identify the independent components of the aliased eye signals [33, 34]. Therefore, the independent component of the mixed eye in each independent component can be judged according to the correlation coefficient of the overlapped eye signals, so as to realize the function of automatically eliminating eye artifacts
3. Removal of bad block. The prelatency and postlatency can be selected according to the actual needs, and the range of refractory is usually chosen from -200 ms to 500 ms. The EEG amplitude is over ±100 *μ*v, and the EEG waveform in the range of refractory value will be ignored when it is analyzed
4. Epochs and averaging. The software of Curry-7 can be used to epochs and average the EEG data of different events. These different events can be distinguished by labels. The interval setting is very important in the epochs and averaging. According to the stimulus interval, we can set pretime (the starting position generally is 10%-20% of stimulus interval) and postlatency (the ending position, the total time is not more than the stimulus intervals, covers the latency of the studied components but does not cover the baseline of the next event segmentation). The specific calculation is shown in the following equation:

(1) $$\begin{array}{c} x_{ij}=\frac{1}{n}\overset{n}{\underset{j=1}{\sum }}x_{ij} \end{array}$$

In equation (1), *j* represents a specific experiment, the sum of the times of the experiment is *n* times, and *i* represents the specific times in the experiment.

(5) Filtering parameters and referencing. Different filtering methods should be chosen according to the research direction. In our experiments, we chose low-pass 30 Hz filtering. This method can better remove the effect of high-frequency noise on EEG. We chose all electrodes in average as the reference. We process and visualize the EEG data by the above methods and through feature extraction and analysis to show our results

### 2.2. Experimental Design

This study has invited 36 volunteers to participate in the experiments, in which males and females are equal in number. The subjects are undergraduate or graduate students with an average age of 21 years old. The subjects are selected according to the psychological experiment standard, healthy, right handed, and so on, and all the subjects voluntarily participated in these experiments. The subjects receive a certain reward after completing these experiments.

**Figure 2:**
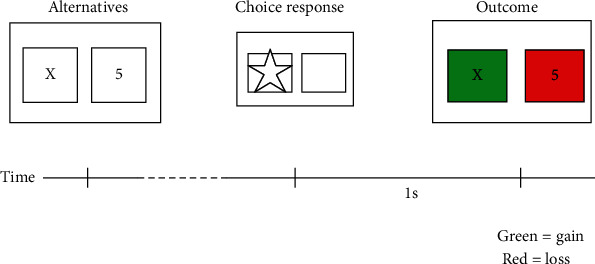
The experiments of game decision-making.

The schematic diagram of the experiments is shown in Figure 2. In the experiments, the participants view two squares without color, each of which contains a different mark, where the left square contains the numeral *X* and the right square contains the numeral 5. The value of *X* will vary with the experiments. The squares will turn red or green when the participants make a choice after a second. If the chosen squares turn red, it means the loss amount of money; if the squares turn green, it means gains. The squares turn red or green with the same probability.

The subjects participate in three experiments, and every experiment lasted for about 15 minutes. It is given sufficient time to rest and relax for the subjects during each interval. We perform three experiments, the value of *X* is equal to 25 in the first experiment, and the value of *X* is 35 and 50 in the second and third experiments, respectively. Our experiments look like a gambling task. Here, we introduce a reward parameter *E*, which is equal to the value of the left square divided by the value of the right one. The parameter *E* provides a systematic measure of rewards and punishments. In our first experiment, the parameter *E* is 5, in the second and third, the parameter *E* is 7 and 10, respectively. In particular, the parameter *E* also reflects the degree of risk. When the parameter *E* is in a larger range, it shows a higher risk level at this time.

Our experiments can control different risk levels through the parameter *E*, which can collect different EEG signals according to the risk levels. Previous studies are conducted only in one aspect of the experiments and do not control the risk levels. Because the different risk levels will affect the choice of the subjects' strategies, our experiments can better discard the influence of risk preference and risk aversion on the experimental results.

In the experiments, the participants can choose different squares by pressing the corresponding button. When the participants click the left mouse button, it means that they chose the square on the left, and when they click the right mouse button, it means they chose the right square. One second after making a choice, the color of the squares will turn red or green. If the selected square turns green, it represents gains and the red means losses. The squares which participants do not choose turn red or green at the same time. For example, when the participants choose the square on the right, it means that the stake is 5 yuan (RMB). The Renminbi (RMB) is the legal tender of China. If the chosen square turns green, it indicates a gain of 5 yuan. If the chosen square turns red, it indicates a loss of 5 yuan. The total revenue of the participants is irrelevant to the unselected square whether it turns red or green. When the outcomes (the square turns red or green) are presented, the participants can see not only the gains or losses which he selected but also the unselected squares. All the squares turn red or green with the same probability, but the participants do not know that. It lasted for about 3 seconds for each choice, and in every 15 minutes' experiment, the subjects selected 300 times. We analyze EEG data of epoch/averaging according to their choices, so our experimental samples are considerable. The final score will determine the revenue of the participants, so that the subjects can be more realistic to raise the credible EEG signals. We collect the EEG signals of the subjects in the entire experiments.

## 3. Results

### 3.1. Before Decision-Making

**Figure 3:**
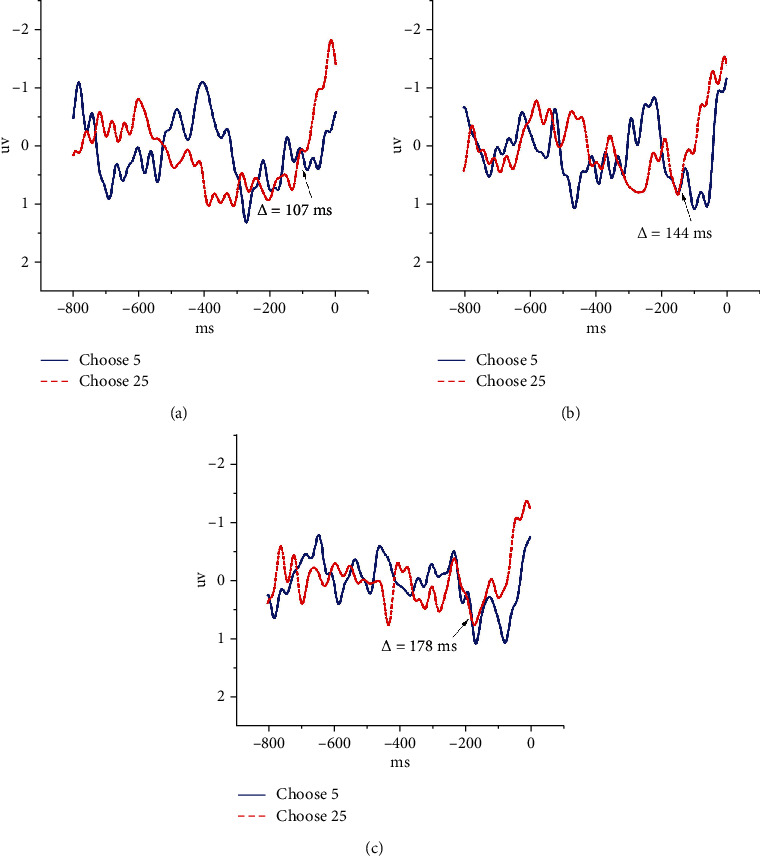
The EEG at Fz in every experiment before making the decision. The PT increases with the parameter *E*. (a) In the first experiment (*E* = 5), the PT is 107 ms. (b) In the second experiment (*E* = 7), the PT is 144 ms. (c) In the third experiment (*E* = 10), the PT is 178 ms.

The MFC is a major area in decision-making, which is reflected by not only the outcomes after the decision-making but also the whole process of decision-making. Here, we study the EEG in the MFC based on the strategy of preanalysis before the decision-making. As shown in Figure 3, we find that the EEG is different at Fz before the subjects make different choices. It means that when the subjects are going to choose the different square, the EEG will be different.

As shown in Figure 3, the abscissa represents the time series in milliseconds, and the 0 ms represents the moment to make a decision. The ordinate is expressed as the magnitude of the voltage value in microvolts. The EEG of different decisions is completely separated around 100 ms before decision-making, and we call this time prestimulation time (PT). The red line represents the average EEG waveform for all trials in which the participants will choose the square on the left (big bet *X*). The blue line corresponds to those trials in which the participants will choose the square on the right (small bet 5).  Figure 3(a) shows *E* = 5 in the first experiment, Figure 3(b) represents *E* = 7 in the second experiment, and Figure 3(c) indicates *E* = 10 in the third experiment.

As shown in Figure 3, we find that there is a big difference in the EEG at the Fz around 100 ms before the decision-making. When the subjects are going to choose the squares on the left (they will choose a big bet), the negative-polarity ERP is higher than those who choose a small bet at the Fz around 100 ms before the decision-making. Moreover, the time when the EEG is clearly separated shows a trend of gradual advance with the increase of the reward parameter *E*. There is a last intersection of the two lines which represent the EEG when the subjects choose different squares in the three experiments. Specifically, the PT in the first experiment (*E* = 5) is 107 ms before making the decision, while in the second experiment (*E* = 7), the PT is 144 ms, and in the third experiment (*E* = 10), the PT is 178 ms. PT is obtained on average by all the subjects in the experiments.

When people make decisions, they always tend to choose what they can get gains. When the participants choose the square on the left, it means that they are making a big bet, which shows that the participants have a higher expectation for the outcome. In contrast, when the participants make a small bet, it means that he has a lower expectation for the outcome. Because of the participants with different expectations of the outcomes, the EEG of the subjects in the MFC are also different. When they have greater expectations for what they choose, they will produce a larger negative-polarity ERP than those who have lower expectations in this brain area. In our three experiments, the participants have the same expectations when they choose the square on the right, but when they choose the square on the left, they will have different expectations, and the participants will have the highest expectations when they choose the left square in the third experiment (where the *E* = 10). We find that the negative-polarity ERP produced at the Fz can be separated earlier from the different expectations. Specially, in the first experiment, the PT is 107 ms; correspondingly in the second and third experiments, the PT is 144 ms and 178 ms. At the same time, we can say that the EEG knows earlier about what we are going to do than ourselves. In fact, EEG has leaked some of our minds in a certain extent. As shown in Table 1, PT has statistically significant differences in the values of three experiments (*P* < 0.01).

The PT indicates an excitation time before stimulation and also shows a degree of expectation of the results, reflecting people's psychological and cognitive activities. Unlike other ERP such as P300, the PT is produced by active spontaneous stimulation of the subjects, which has potential value in the study of cognitive function and mental illness. For example, we can obtain the range of PT value of normal people through experiments and take this index as a reference for the evaluation of cognitive impairment and mental diseases.

The PT has important application and reference value in the research and analysis of behavioral decision-making. It is a kind of ERP triggered by the expectations of the outcomes. It not only intuitively reflects the expectations of the results but also reflects the types of risk choices in the behavioral decision-making. If the PT is larger, it reflects the risk preference type, and vice versa. Therefore, we can judge whether the subject is risk preference type or risk aversion type through this index. For example, the PT can be used as an indicator to assess a person's risk appetite, which can be used to effectively predict risk appetite and make judgments about future behavioral decisions through this indicator.

**Figure 4:**
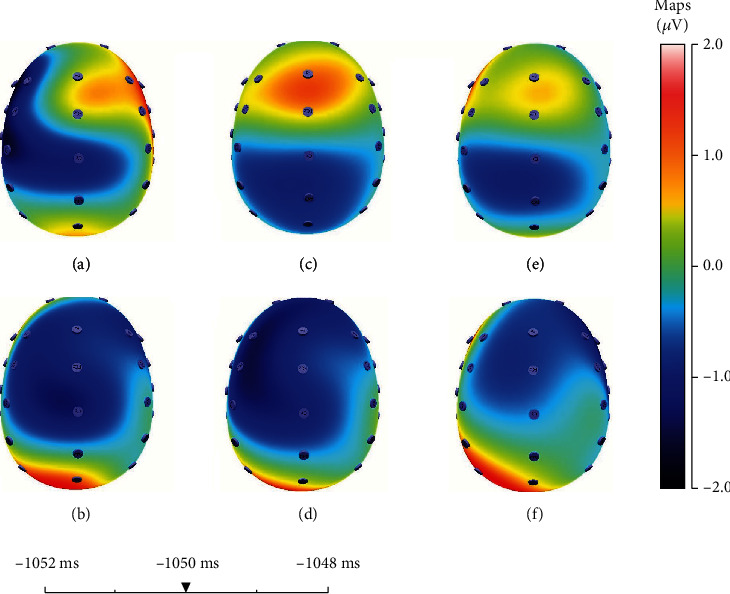
The effects of different expectations on the various regions of the brain 50 ms before making a choice. The red region tends to show obvious positive characteristics, and the blue region tends to show negative ones. (a) The first experiment where *E* = 5, and the subjects will choose 5. (b) The first experiment where *E* = 5, and the subjects will choose 25. (c) The second experiment where *E* = 7, and the subjects will choose 5. (d) The second experiment where *E* = 7, and the subjects will choose 35. (e) The third experiment where *E* = 10, and the subjects will choose 5. (f) The third experiment where *E* = 10, and the subjects will choose 50.

In addition, we can find that the absolute value of the negative EEG produced between different expectations at 50 ms before the decision-making reaches a maximum value (see Figure 4); that is to say, the absolute value of EEG produced by different expectations is largest at this time. Therefore, we investigate the effects of different expectations on all regions of the brain at this time (50 ms before the decision-making), to further explore the impact of behavioral decisions on EEG.

As shown in Figure 4, the entire brain area produces a more obvious negative-polarity ERP when the subjects have greater expectations in the same experiment, and these negative-polarity ERPs are mainly concentrated in the forehead frontal region. When people choose different bets, they will have different expectations for the outcomes, so it will have different effects on the EEG. Before making different decisions, the entire brain region has shown a distinctly different characteristic in EEG. Therefore, we can make a prediction of the upcoming decisions based on these characteristics. We have further confirmed that EEG leaks out the decisions we are going to make by studying the EEG throughout the entire brain region.

### 3.2. After Decision-Making

The medial frontal cortex (MFC) is the main part of the brain involved in decision-making, which controls the individuals' social behaviors, emotions, and decision-making behaviors [35–37]. The Fz lead is mainly used to collect the EEG signals in MFC [38–40]. Through the analysis of the EEG signals in the Fz lead, we find that the EEG signals varied with the different outcomes, especially in the gains and losses cases. Moreover, the experimental results are quite different with the difference of the reward parameter *E*.

**Figure 5:**
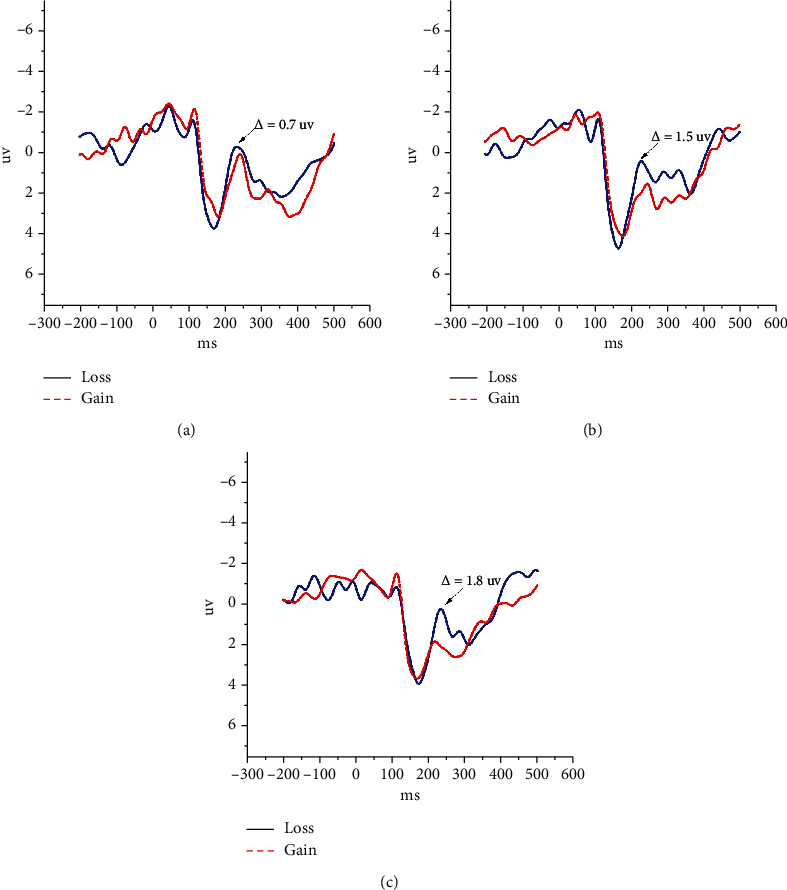
The EEG at Fz in every experiment after making the decision. The EEG showed negative-polarity ERP obviously in the 232 ms at Fz. There is an obvious absolute value of negative-polarity ERP produced by losses and gains. With the increase of *E*, the absolute value increased clearly. (a) In the first experiment (*E* = 5), the absolute value of waves produced by losses and gains is 0.7 *μ*v in the 232 ms. (b) In the second experiment (*E* = 7), the value is 1.5 *μ*v. (c) In the third experiment (*E* = 10), the value is 1.8 *μ*v.

As shown in Figure 5, the abscissa represents the time series in milliseconds, the 0 ms represents the moment to make a decision, and the ordinate is expressed as the magnitude of the voltage value in microvolts. The EEG shows negative-polarity ERP obviously in the 232 ms at Fz. The red line corresponds to the average EEG waveform of all trials in which the participants gain. The blue line represents those trials in which the participants lose. There is an obvious absolute value of negative-polarity ERP produced by losses and gains in 232 ms. With the increase of *E*, the absolute value increased clearly. The results are obtained by averaging from 36 samples.

Figure 5 shows that the EEG generated by the losses at the Fz is higher than that generated by the gains around 232 ms after the outcome is presented, and the results are shown in the three experiments. There is an absolute value that will be generated by the negative-polarity ERP between the losses and gains when the outcomes appear in 232 ms. As shown in Table 2, the absolute value is 0.71 ± 0.11 *μ*v in the first experiment (*E* = 5), 1.52 ± 0.17 *μ*v in the second experiment (*E* = 7), and 1.83 ± 0.23 *μ*v in the third experiment (*E* = 10). In the three experiment, the EEG produced by loss and gain showed statistically significant differences (*P* < 0.05). The absolute value also has statistically significant differences in three experiments (*P* < 0.05).

The results suggest that the MFC is a major part of the brain involved in decision-making and the EEG in this area is influenced by economic losses or gains obviously. Moreover, the impact of losses is stronger than that of the gains on the EEG in this region. The above results are from the Fz to analyze the impact of losses and gains on the brain. However, what will be the impact of losses and gains on the EEG in all areas of the brain when the outcomes appeared after 232 ms. In order to deal with this problem, we study the effects of losses and gains on the EEG in the whole regions of the brain.

**Figure 6:**
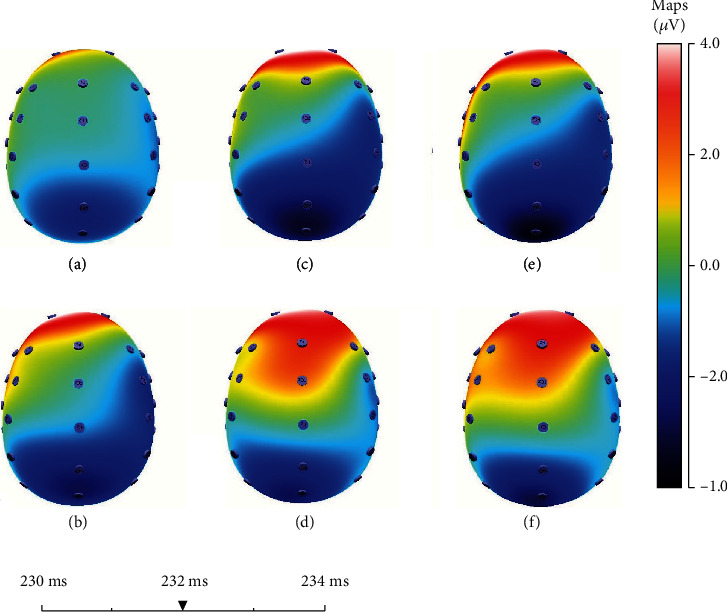
The effects of losses and gains on the EEG in the whole regions of the brain when the outcomes appeared after 232 ms. The red region tends to show obviously the characteristics of positive-polarity ERP, and the blue region tends to show negative-polarity ERP. (a, c, and e) Represent the losses; (b, d, and f) show the gains. (a, b) indicate that *E* = 5; (c, d) mean that *E* = 7; (e, f) show that *E* = 10.

As shown in Figure 6, we find that the EEGs caused by the losses are significantly more negative than the gains at Fz when the outcomes are presented around 232 ms. This phenomenon is obvious with the increase of *E*, which can verify the results we mentioned above. We also find that due to the losses, the hindbrain area produced a more significant negative-polarity ERP; and due to the gains, the forebrain area produced a more significant positive-polarity ERP.

## 4. Discussion

### 4.1. The Understanding of the Meaning of the Results

In our study, we concern more about the effects of prior decision-making on EEGs. In our experiments, we take into account three different-size scale experimental models (*E* = 5, 7, and 10) and carry out a series of experiments. By analyzing the event-related potential (ERP) of the experimental data, we find that the difference between gains and losses on the negative wave increased with the scale of the parameter *E*, and the losses have a stronger effect on the prefrontal region than gains, suggesting that the prefrontal region is the main part of the brain involved in decision-making and has a greater impact on economic gains and losses. In addition, the EEG results in the experimental results indicate that stimuli due to loss produced significant negative waves in the hindbrain region, while stimuli due to gain produced significant positive waves in the forebrain region, indicating that the forebrain regions responded more significantly to gains, while the hindbrain regions respond more significantly to losses. The anterior frontal region of the midbrain is the main area involved in decision-making not only in terms of response to outcomes but also in terms of the impact on this area before making different decisions. Experimental results show that the greater the risk taken in a decision, the more pronounced the negative wave in the anterior frontal region, and the greater the expectation of the benefit, the earlier the negative wave is generated.

Because of the different effects of gains and losses on brain waves, we can use the results of this experiment to determine the specific response of gains and losses on a person's brain waves, to determine the difference with others on that experiment, and thus to obtain the specific activity state of brain waves. Furthermore, the brain waves generate for different risks and expectations before decision-making can be used to determine a person's risk appetite or to make a prediction about the upcoming decision. Moreover, the results of our study provide a reliable theoretical basis for the future researches on behavioral decision-making. These findings may be helpful for the study of behavioral decision-making, such as lie detecting or decision prediction, involving the prediction of gains or losses relative to the status quo.

### 4.2. Comparison with Previous Studies

In reference [5], the authors experimentally demonstrate that human behaviors can be more powerfully influenced by conditioned reinforcers. However, they have not provide a system for conditional enhancement of reward and penalty mechanisms. In our study, we have introduced a reward parameter *E* which provided a quantitative indicator in the degree of risk and demonstrated through a series of experiments that the EEG signal in the prefrontal region of the brain is affected differently with different reward and punishment parameters *E*. In the study on EEG, we find that the impact of losses is stronger than that of the gains on the brain, and our results show that the EEG waves generate by the losses are more negative than the gains after the outcomes, which also further validated the conclusions of previous psychological studies.

It is shown that previous studies have paid more attention to the effects of stimuli produced by different outcomes on EEG [6, 14]. These studies have allowed us to understand behavioral decisions more profoundly from EEG. In particular, the impacts of economic losses and gains on the brain enable us to better understand the behaviors in decision-making. It follows that MFC may contribute to mental states in which participants make higher level decisions, including economic choices [16]. In this study, our results not only show that the MFC is the main part of the brain involved in decision-making but also show that the impact of losses on the brain is more intense in this area.

Most of the previous studies have studied the EEG generated by the subjects after the stimulation [7, 15, 16], but there are few studies on the EEG produced before stimulation. At the same time, it is rare to propose that the EEG can be used to predict behaviors. In fact, the stimulation of EEG has begun before decision-making, and analyzing the EEG before the decision-making will produce a significant meaning for the prediction of the behavioral decision-making. However, most previous studies have ignored this point [11, 17, 19]. In our study, we concern more about the effects of prior decision-making on EEGs, people tend to have different expectations of the outcome when making decisions, and the larger of expectations, the more pronounced the negative waves in the frontal lobe of the brain. The results of the study indicate that EEGs can predict behavioral decisions.

A limitation of the present study is that the subjects in our experiments are all young students and the number of samples is not abundant enough. Future studies may include increasing the number and diversity of subjects. When the number of samples is rich, the samples can extract more detailed features. While the samples can be trained and classified better, it will make a more accurate prediction for the upcoming decision of the individuals.

## 5. Conclusion

In this paper, our results show that the MFC is the main part of the brain involved in decision-making, and the impact of losses on the brain is more intense in this area. It shows that due to the impact of the losses, the hindbrain area produces a more significant negative-polarity ERP, and due to gains, the forebrain area produces a more significant positive-polarity ERP. We find that the EEGs are clearly distinguishable before making different decisions. Because of the influences of different expectations, the EEGs are also different. It will generate significant negative-polarity ERP in the entire brain region when the participant has a greater expectation for the outcomes, and these ERPs are mainly concentrated in the forebrain region. In fact, this suggests that EEGs have different characteristics before making decisions, and these different features can be detected before the outcomes are presented. Therefore, we can make a prediction for the upcoming selections on the basis of these distinguished features. At the same time, our work also provides a theoretical basis for using EEG to predict behavioral decisions. In addition, the proposed PT also provides a new basis for the study of cognitive function and neurological diseases. We believe that this indicator will play a greater role in future researches, especially in the medical field.

## Figures and Tables

**Table 1:** The PT in different experiments.

Experiments	*E* = 5	*E* = 7	*E* = 10
PT	107 ± 8.8 ms	144 ± 9.6 ms	178 ± 10.3 ms

**Table 2:** The voltage amplitude of gains and losses after making the decision in different experiments.

Experiments	*E* = 5	*E* = 7	*E* = 10
Loss	−0.34 ± 0.07 *μ*v	+0.52 ± 0.06 *μ*v	+0.32 ± 0.09 *μ*v
Gain	+0.37 ± 0.05 *μ*v	+2.04 ± 0.08 *μ*v	+2.15 ± 0.07 *μ*v
Time	230 ± 11 ms	232 ± 12 ms	235 ± 15 ms
∆	0.71 ± 0.11 *μ*v	1.52 ± 0.17 *μ*v	1.83 ± 0.23 *μ*v

## Data Availability

The data used to support the findings of this study are available from the corresponding author upon request.
